# Genome Sequencing of 18 Francisella Strains To Aid in Assay Development and Testing

**DOI:** 10.1128/genomeA.00147-15

**Published:** 2015-04-30

**Authors:** Shannon L. Johnson, Hajnalka E. Daligault, Karen W. Davenport, Susan R. Coyne, Kenneth G. Frey, Galina I. Koroleva, Stacey M. Broomall, Kimberly A. Bishop-Lilly, David C. Bruce, Olga Chertkov, Tracey Freitas, James Jaissle, Jason T. Ladner, C. Nicole Rosenzweig, Henry S. Gibbons, Gustavo F. Palacios, Cassie L. Redden, Yan Xu, Timothy D. Minogue, Patrick S. Chain

**Affiliations:** a Los Alamos National Laboratory (LANL), Los Alamos, New Mexico, USA; b United States Army Medical Research Institute of Infectious Diseases (USAMRIID) Diagnostic Systems Division (DSD), Fort Detrick, Maryland, USA; c Naval Medical Research Center (NMRC)-Frederick, Fort Detrick, Maryland; d Henry M. Jackson Foundation, Bethesda, Maryland, USA; e Center for Genome Sciences (CGS), United States Army Medical Research Institute of Infectious Diseases (USAMRIID), Fort Detrick, Maryland, USA; f United States Army Edgewood Chemical Biological Center (ECBC), Aberdeen Proving Ground, Aberdeen, Maryland, USA

## Abstract

Francisella tularensis is a highly infectious bacterium with the potential to cause high fatality rates if infections are untreated. To aid in the development of rapid and accurate detection assays, we have sequenced and annotated the genomes of 18 F. tularensis and Francisella philomiragia strains.

## GENOME ANNOUNCEMENT

Francisella tularensis is the causative agent of tularemia, a zoonotic infection that is mainly transmitted to humans through arthropod bites, direct contact with infected animals, or the inhalation/ingestion of contaminated materials (dust, food, or water). These Gram-negative, nonmotile, facultatively intracellular bacteria are highly infectious (1). The low infectious dose (10 to 50 organisms) and previous weaponization attempts have led to the placement of F. tularensis on the U.S. Centers for Disease Control and Prevention category A biothreat agent list (2–4). In 1970, a World Health Organization (WHO) expert committee reported that if sufficient quantities of F. tularensis were dispersed over a metropolitan area with 5 million people, it could result in approximately 250,000 acute febrile nonspecific illnesses 3 to 5 days postrelease and 19,000 deaths (5).

Francisella is endemic to the United States and resides within small mammals (mice, voles, rats, squirrels, rabbits, and hares) that serve as natural reservoirs. There are three subspecies of F. tularensis: F. tularensis subsp. tularensis, which can be highly virulent, with a mortality rate of 30 to 60% (without treatment), and the occasional human pathogens F. tularensis subsp. *holartica* and F. tularensis subsp. *mediasiatica*. The close phylogenetic relationship of highly virulent strains to opportunistic pathogens and near neighbors has caused taxonomic and strain differentiation difficulties.

Here, we present the annotated genome assemblies for all but one strain listed in the Standard Method Performance Requirements (SMPR) as derived by the Stakeholder Panel on Agent Detection Assays (SPADA) (FTTN10 is not currently maintained in a publicly available culture collection). The 18 genome sequences presented here were assembled to finished or improved high-quality draft (IHQD) status (6).

The draft genome assemblies included two or more data sets (data types and coverages are listed in the NCBI records): Illumina (short- and/or long-insert paired data), Roche 454 (long-insert paired data), and PacBio long reads. The short- and long-insert paired data were assembled in both Newbler and Velvet (7) and computationally shredded into 1.5-kbp overlapping shreds. If the PacBio coverage was ≥100×, the data were assembled using HGAP (8). All data were additionally assembled in AllPaths (9). The consensus sequences from both HGAP and AllPaths were computationally shredded into 10-kbp overlapping pieces. All shreds were integrated using Phrap. Possible misassemblies were corrected and repeat regions verified using in-house scripts and manual editing in Consed (10–12). When combined with the long-insert (~8 to 10-kb) Illumina data, the HGAP assemblies of recent PacBio data are generally capable of reconstructing the ~30-kb pathogenicity island repeat found in Francisella genomes. These methods resulted in a finished quality genome for 17 of the 18 isolates (Table 1) (6). Each genome assembly was annotated using an Ergatis-based (13) workflow with minor manual curation.

**TABLE 1:** Listing of Francisella isolate genomes released to NCBI as listed in the SMPR by the Association of Analytical Communities

Organism name	Panel^*a*^	AOAC name^*b*^	GenBank accession no.	Quality^*c*^	Assembly size (bp)	No. of plasmids
F. tularensis subsp. tularensis						
WY96	I	FT9	CP010103	Finished	2,047,033	1
Schu_S4	I	FT4	CP010290	Finished	1,892,789	0
Scherm	I	FT8	CP010287	Finished	1,890,922	0
NIH B-38	I	FT1	CP010115	Finished	1,862,444	0
F. tularensis subsp. *novicida*						
U112	E	FTNN9	CP009633	Finished	1,910,592	0
F6168	E	FTNN8	CP009352–CP009353	Finished	1,927,240	1
D9876	E	FTNN7	CP009607	Finished	1,870,206	0
F. tularensis subsp. *holartica*						
VT68	I	FT6	CP010288	Finished	1,893,733	0
LVS	I	FT2	CP009694	Finished	1,892,177	0
JAP	I	FT5	JUJU00000000	IHQD (2)	1,907,159	0
FTT_1	I	FT3	CP009693	Finished	1,892,758	0
425	I	FT7	CP010289	Finished	1,894,186	0
F. philomiragia						
O#319l	E	FTNN1	CP010019	Finished	2,017,400	0
O#319-067	E	FTNN4	CP009436–CP009437	Finished	2,049,711	1
O#319-036	E	FTNN3	CP009442–CP009443	Finished	1,924,061	1
O#319-029	E	FTNN2	CP009342–CP009343	Finished	2,044,934	1
GA01-2801	E	FTNN6	CP009444–CP009446	Finished	2,033,714	2
GA01-2794	E	FTNN5	CP009440–CP009441	Finished	2,152,054	1

a I, inclusivity strain; E, exclusivity strain.

b AOAC, Association of Analytical Communities.

c Quality refers to the quality of the genome assembly, as defined by Chain et al (6). The number of contigs for the IHQD assembly is listed in parentheses.

The genome assemblies range from 1.86 to 2.15 Mb (Table 1), with one chromosome and up to two plasmids. As expected for the genus, the G+C% was low, ranging from 32 to 33%, and genomes have between 1,769 and 2,107 coding sequences.

### Nucleotide sequence accession numbers. 

The accession numbers for all 18 genome sequences are listed in Table 1.
